# Acute Spontaneously Resolving Pulmonary Vasculitis: A Case Report

**DOI:** 10.1155/2012/706838

**Published:** 2012-11-20

**Authors:** James B. Geake, Graeme Maguire

**Affiliations:** ^1^Department of Respiratory and Sleep Medicine, Monash Medical Centre, Melbourne, VIC 3206, Australia; ^2^Cairns Clinical School, School of Medicine and Dentistry, Faculty of Medicine, Health and Molecular Sciences, James Cook University, P.O. Box 902, Cairns, QLD 4870, Australia; ^3^Baker IDI Central Australia, P.O. Box 1294, Alice Springs, NT 0870, Australia

## Abstract

This is the first description that we are aware of describing the spontaneous resolution of an acute pulmonary vasculitis, possibly secondary to microscopic polyangiitis. Haemoptysis is a common symptom for patients presenting to primary and tertiary referral centres, and pulmonary vasculitis is one of a variety of aetiologies that should always be considered. The pulmonary vasculitides are difficult diagnostic and management problems. They are encumbered by a relative paucity of level 1 evidence addressing their diagnosis, classification, and treatment. This is therefore an important paper to publish because it adds to the global breadth of experience with this important clinical condition.

In July 2010 a 43-year-old female presented with a 24-hour history of 30 mL of frank haemoptysis. There were no symptoms suggestive of a respiratory infection. She worked as an office manager and had no relevant occupational or environmental exposures. She had ceased smoking ten years previously having accumulated a ten-pack-year history. She had hypertension treated with amlodipine and perimenstrual migraines. Both parents and her brother were alive without any significant medical diseases.

On presentation peripheral arterial oxygen saturation was 85% on room air, respiratory rate was 24 per minute, blood pressure was 200/100, and temperature was 38.5°C. She remained afebrile thereafter. An ECG demonstrated sinus rhythm, 100 beats per minute. A chest X-ray demonstrated bilateral perihilar infiltrates. A CTPA confirmed diffuse bilateral alveolar infiltrates (Figure 1). No vascular filling defects were identified. Haemoglobin was 99 g/L (115–160). MCV (mean cell volume) was 67 fL (81–96), and the film was suggestive of iron deficiency. White cell count was mildly elevated at 137/nL (3.5–11). Other inflammatory markers were also mildly elevated. ESR (erythrocyte sedimentation rate) was 29 mm (<15). CRP (C-reactive protein) was 68.1 mgl/L (<8). Creatinine was 65 *μ*mol/L (50–100). Urea was 48 *μ*mol/L (2.7–7.8). Electrolytes were within normal limits. INR (international normalised ratio) was 1.1 (0*·*9–1.2). APTT (activated partial thromboplastin time) was 28 seconds (25–34). Testing for antiglomerular basement membrane, antiuclear cytoplasmic antibodies (cANCA and pANCA), antinuclear antibodies, and extractable nuclear antigens (ENA) were all negative. Rheumatoid factor was <10 IU/mL (<30). Several sputum samples were sent. No pathogens were identified on standard bacterial and mycobacterial cultures. There was no cytological evidence of malignancy. Urinalysis was normal. Phase contrast microscopy was not performed as the red cell count on several specimens was within normal limits. Albumin/creatinine ratio was 17.7 g/mol creatinine (<3.5). The patient was treated with benzylpenicillin and doxycycline, and there was improved, but persisting haemoptysis. On the third day the patient proceeded to fibre optic bronchoscopy where no source of bleeding or endobronchial abnormality was identified. A surgical lung biopsy was subsequently performed. At the operation blood was noted within the pleural space, the surface of the lung was oozing fresh blood, and the parenchyma was boggy. A wedge biopsy was taken from the right middle lobe. Microscopy demonstrated a widespread interstitial inflammatory process centred on capillary sized vessels. There were associated interstitial haemorrhage and fibrin deposition. Occasional neutrophils and eosinophils were both observed. The disease process was variable in intensity with propensity for different expression on either side of an interlobular septum. No granulomata were identified. Special stains were negative for fungal hyphae and *Pneumocystis* species (Figures 2 and 3). A diagnosis of small vessel pulmonary vasculitis was made, thought to be most consistent with microscopic polyangiitis.

The patient made an uneventful recovery from the surgical procedure and the haemoptysis appeared to rapidly spontaneously settle. Induction immunosuppression with cyclophosphamide and high dose prednisolone was discussed. However, the patient declined. The patient was therefore, discharged three days after the operation with close outpatient surveillance arranged. Repeated clinical reviews with an assessment of lung function, chest radiology, renal function, and urinalysis demonstrated continued complete resolution of pulmonary vasculitis. 12 months after her initial presentation she remained well.

Pulmonary vasculitis should be considered in the differential diagnosis of haemoptysis. Microscopic polyangiitis is a small vessel systemic vasculitis that usually manifests with glomerulonephritis. The lungs are involved in association in approximately 20% to 30% of cases, with diffuse alveolar haemorrhage in approximately 10% to 20% [1, 2]. It is associated with pANCA in 90% of the cases [3]. It is rare for this disease to present with diffuse alveolar haemorrhage in isolation. Treatment recommendations are largely based on historical data demonstrating poor prognosis for patients with pauciimmune glomerulonephritis. No randomised controlled trials have been performed for patients with pulmonary haemorrhage. Many advocate aggressive immunosuppression in this clinical situation with a combination of cyclophosphamide and high dose of prednisolone, with or without plasma exchange [4]. Owing to the toxic effects of this regimen, different strategies have been investigated, and promising disease modifying and biological alternatives continue to emerge, both for induction and maintenance immunosuppression.

Here we described the unusual case of small vessel pulmonary vasculitis with spontaneous remission. Whilst the histology is thought to favour a diagnosis of microscopic polyangiitis, it is possible that an alternate diagnosis was missed, for example, through sampling error. This case highlights the difficulties applying guidelines to rare diseases, particularly when presentations are atypical, as was the case of this patient.

## Figures and Tables

**Figure 1:**
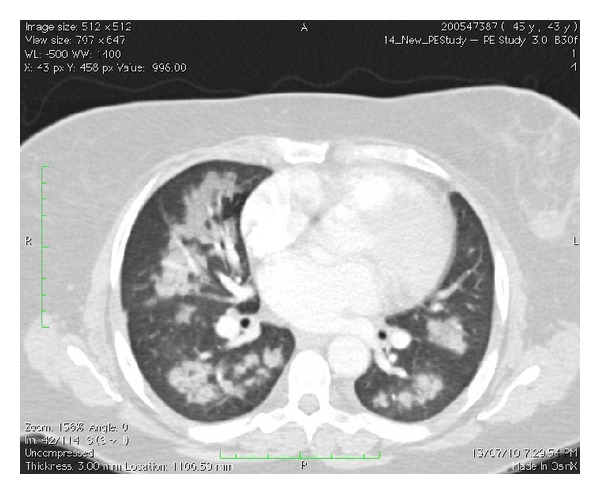
Coronal CT chest demonstrating bilateral perihilar pulmonary infiltrates consistent with diffuse alveolar haemorrhage.

**Figure 2:**
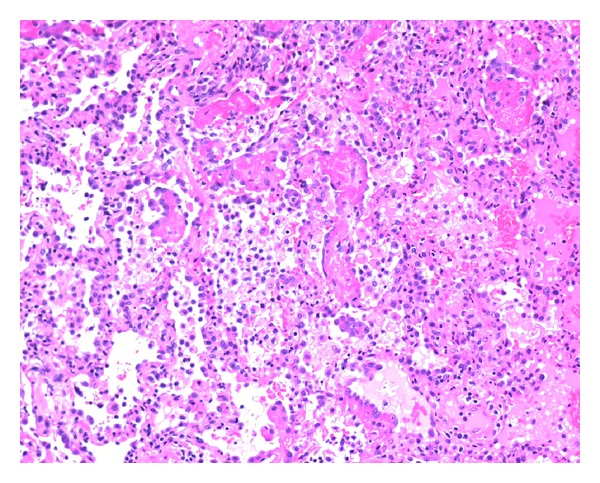
Low power magnification of hematoxylin and eosin stained lung biopsy demonstrating a pauci-immune small vessel vasculitis.

**Figure 3:**
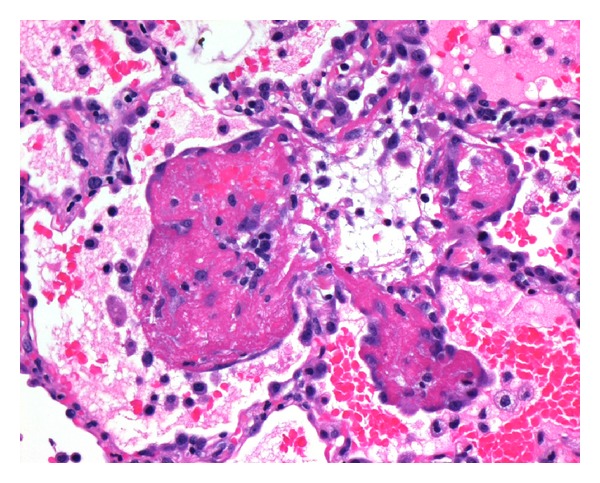
High power magnification of hematoxylin and eosin stained lung biopsy demonstrating a pauci-immune small vessel vasculitis.

## Abbreviations

MCV: Mean cell volume
ESR: Erythrocyte sedimentation rate
CRP: C-reactive protein
INR: International normalised ratio
ENA: Extractable nuclear antigens
ANCA: Antinuclear cytoplasmic antibodies.
